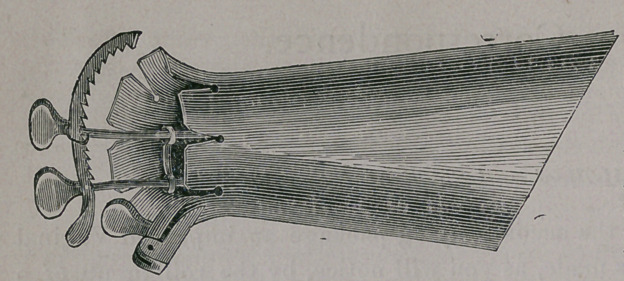# A New Speculum

**Published:** 1878-06

**Authors:** S. G. Dorr

**Affiliations:** Buffalo, N. Y.


### Correspondence.

*To the Buffalo Medical and Surgical Journal:*

Please find in the accompanying package an improved Vaginal Speculum. It is made, as you will notice, by the rolling up of a sheet of hard rubber, when heated, in the form of a scroll, with suitable levers and clutches for opening or closing the same.

The one I send you is a large size, too large for the ordinary use of physicians. In the construction of this instrument I have intended that it should possess all the good points of both the cylindrical and bivalvular classes. When closed, as shown by the cut, it is a conical-shaped instrument, smallest at the inner end.

In this condition it is easy of introduction, and the physician can see through it, facilitating very much the finding of whatever he may be in search of.

When the instrument has been properly introduced and the object brought to view, the inner end may be largely expanded, as shown in the cut, thus giving a wider field of view for operations than can be obtained by an ordinary cylindrical Speculum capable of introduction.

When expanded its form prevents its displacement and the physican has the liberty of both hands. The vaginal walls do not close in as with the valvular class.

I had these instruments made by the Buffalo Dental Manufacturing Company some time ago, and have since used a smaller one quite frequently, and like it. Yours,

S. G. Dorr, M. D.,
Buffalo, N. Y.

We have received the instrument represented in the above cuts, and from its easy adjustment and ample exposure of uterine neck judge it often useful in surgical operations as well as in applications to the uterine neck. It is ingenious in its construction, and may be used in all cases, both for diagnosis and treatment, though its chief advantage is full exposure of the neck for surgical operation. Ed.

## Figures and Tables

**Figure f1:**
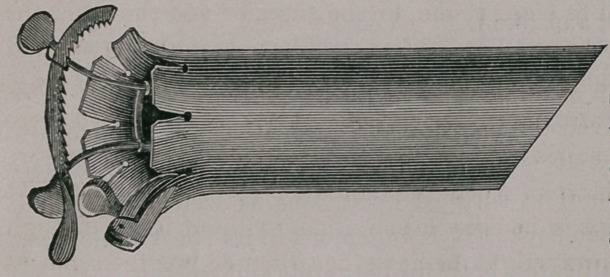


**Figure f2:**
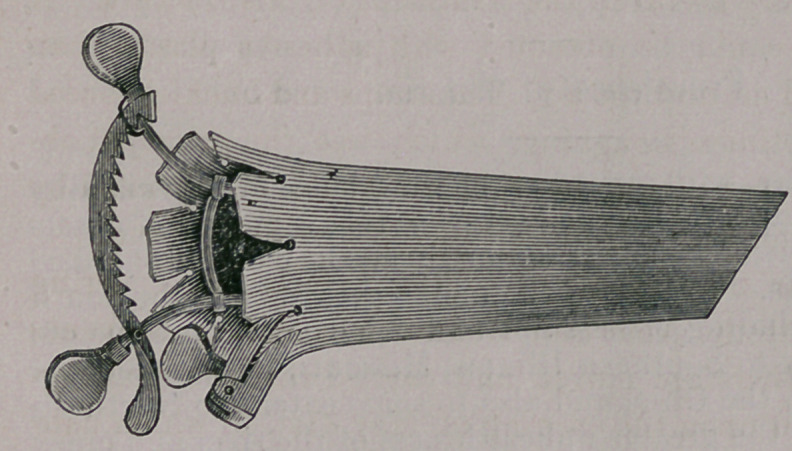


**Figure f3:**